# Leachability and Chemical Profiles of Per- and Polyfluoroalkyl Substances in Electronic Waste Components: Targeted and Non-Targeted Analysis

**DOI:** 10.3390/molecules31030445

**Published:** 2026-01-27

**Authors:** Joshua O. Ocheje, Yelena Katsenovich, Berrin Tansel, Craig P. Dufresne, Natalia Quinete

**Affiliations:** 1 Institute of Environment, Department of Chemistry and Biochemistry, Florida International University, North Miami, FL 33181, USA; joocheje@gmail.com; 2 Applied Research Center, Florida International University, Miami, FL 33174, USA; katsenov@fiu.edu; 3 Civil and Environmental Engineering Department, Florida International University, Miami, FL 33174, USA; tanselb@fiu.edu; 4 Thermo Fisher Scientific, 1400 Northpoint Pkwy, West Palm Beach, FL 33407, USA; craig.dufresne@thermofisher.com

**Keywords:** e-waste, PFAS, waste management, non-targeted analysis (NTA), partitioning behavior

## Abstract

Electronic waste (e-waste) is a growing solid waste stream with largely undisclosed and poorly characterized fluorinated constituents. We evaluated per- and polyfluoroalkyl substances (PFAS) leachability from four e-waste components (phone screens, phone plastics, capacitors, and Lithium-ion batteries) using a 30-day deionized water leaching test. PFAS were extracted by solid-phase extraction using weak anion exchange (WAX) cartridges and analyzed with a liquid chromatography triple-quadrupole mass spectrometer. In addition, the PFAS chemical profiles of e-waste components were characterized by non-targeted analysis. Leachable sums of detected PFAS (∑PFAS) were highest in phone screens (1739–1932 ng·kg^−1^) and phone plastics (1575–2197 ng·kg^−1^) and an order of magnitude lower in Lithium-ion batteries (148–158 ng·kg^−1^) and capacitors (147–243 ng·kg^−1^). Short-chain perfluoroalkyl acids (PFAAs) (e.g., PFBA, PFHxA) and legacy acids (e.g., PFOA, PFNA) were more prevalent in phone screens/plastics, whereas capacitors and batteries showed mixed sulfonate/carboxylate patterns (PFOS, PFHxS, and 6:2 FTS). Although capacitors and Lithium-ion batteries contained essential PFAS with high hazard potential at trace levels, phone screens and phone plastics pose a greater risk per mass due to higher ∑PFAS levels and larger volumes. Non-targeted analysis using Orbitrap Astral revealed CF_2_/CF_2_O homologous trends (confidence levels 2–3) with corroborating targeted findings. These findings highlight the need for PFAS-free alternatives, the disclosure of fluorinated additives, and stronger end-of-life management strategies to prevent PFAS releases from e-waste.

## 1. Introduction

Electronic waste (also known as e-waste) is one of the fastest growing solid waste streams globally, driven by rapid technological advancements, increasing consumer demand, and short product lifespans [1]. Devices such as smartphones, laptops, and household electronics contain a complex array of components, including metals, polymers, ceramics, and adhesives [2]. Among these materials, increasing attention is being drawn to the presence of chemical additives, especially persistent and hazardous substances such as per- and polyfluoroalkyl substances (PFAS) [3,4]. PFAS are synthetic organofluorine compounds used extensively in the manufacturing of electronic components due to their thermal stability, resistance to water, oil, and stains, and unique surface-active properties. In electronics, PFAS are commonly used as surfactants, flame retardants, dielectric fluids, lubricants, and polymer processing aids [5]. However, their resistance to environmental degradation and potential for bioaccumulation raise concerns about the release of PFAS from discarded electronic components into the environment [6].

The growing body of evidence indicates that PFAS are not only present in aqueous firefighting foams and industrial wastes but also embedded in consumer goods, including electronic devices such as Lithium-ion batteries, capacitors, phone screens, and plastic housings, either as functional additives or as processing residues [3,4,5,7]. Over time, in uncontrolled recycling or disposal, including open dumping, informal dismantling, or landfilling, these chemicals may be mobilized into surrounding soils and water systems through leaching. Laboratory-based leaching simulations, which mimic environmental conditions such as rainfall infiltration, acidic media, or landfill leachate, provide critical insights into the potential for PFAS to migrate from solid matrices into liquid phases [3,4,8,9,10,11,12,13]. These simulations are needed for understanding the risks posed by improperly managed e-waste in both developed and developing nations, where e-waste management infrastructure remains inadequate.

Targeted PFAS analysis focuses on a select number of known PFAS (typically 20–40 compounds), which represent only a small fragment of the thousands of PFAS identified to date [14]. However, because the chemical profiles of most additives used in the manufacturing of electronic devices are not fully disclosed, the specific type of PFAS or precursors present in these products remain unknown. Non-targeted analysis (NTA) workflows allow for the discovery and screening of a much broader spectrum of fluorinated substances, including emerging and previously unreported compounds for complex matrices, such as e-waste, where proprietary chemical formulations and manufacturing variability make comprehensive identification challenging [15]. Using a non-targeted approach can resolve component-specific PFAS fingerprints and reveal a chemical diversity that is not completely captured by conventional targeted methods. When combined with leaching tests, these approaches enable the comprehensive characterization of PFAS levels and environmental mobility in solid waste matrices.

Despite growing concern, research on PFAS in e-waste remains relatively limited, particularly compared with traditional contaminants such as heavy metals and brominated flame retardants [16]. Current regulatory frameworks and waste treatment protocols often overlook the presence of fluorinated compounds in electronics [17]. Moreover, there is limited knowledge of how different components, such as Lithium-ion batteries, capacitors, phone screens, and plastic casings, differ in their PFAS content, chemical profiles, and leaching behaviors. This knowledge gap hinders policymakers, recyclers, and environmental agencies in implementing effective control strategies or remediation technologies.

With the increased volume of electronic waste and the recognition of PFAS as contaminants of emerging concern, it is critical to understand the presence, levels, and fate of PFAS in electronic components. A few studies have reported PFAS in e-waste [16,18,19]. This limited data on the types of PFAS used and their leaching potential poses a significant risk to both the environment and public health. A previous study by our team identified and quantified 21 PFAS in leachates from e-waste components from computers, including cables, keyboards, e-boards, and monitors [3]. Thirty-day batch leaching tests showed that PFAS concentrations decreased in the following order, cables > keyboards > e-boards > monitors, while potential toxicity was highest in keyboards, followed by cables, e-boards, and monitors. The contribution of e-waste to PFAS release in the environment remains largely overlooked in global waste and water quality management due to limited and non-representative data and a lack of standardized monitoring procedures. This study addresses this gap by conducting leaching tests and the targeted and non-targeted PFAS screening of selected e-waste components to assess the potential risks of e-waste as a PFAS source.

## 2. Results and Discussion

### 2.1. PFAS Content in E-Waste Components

A total of 16 PFAS were detected among the 40 targeted PFAS across all e-waste components; however, the distribution of individual concentrations and total PFAS concentrations differed markedly by the e-waste component. Table 1 presents the results expressed in ng·kg^−1^. The list of all 40 PFAS analyzed in this study is provided in Table S1. About 44% of the compounds were detected in all e-waste components, while 56% were detected in at least one component. The most prevalent compounds were 6:2 FTS, PFOA, PFOS, PFNA, PFDoA, and PFHpA, with detection frequencies of 75–100%. In terms of concentrations detected, PFBA, PFOA, PFNA, and PFHxA were the primary contributors to the total sum of PFAS (∑PFAS) across most components. The ∑PFAS in phone screens was the highest (1739–1932 ng·kg^−1^), followed by phone plastics (1575–2197 ng·kg^−1^), while Lithium-ion batteries and capacitors were an order of magnitude lower (148–158 and 147–243 ng·kg^−1^, respectively).

A one-way ANOVA showed that the e-waste components significantly influenced total PFAS concentrations (F_3,4_ = 35.01, *p* = 0.0025), indicating substantial differences in PFAS burdens among the four components examined. Tukey’s HSD post hoc tests show that mobile phone screens and phone plastics had significantly higher total PFAS concentrations than Lithium-ion batteries and capacitors (all *p* < 0.01). No significant differences were observed between mobile phone screens and phone plastics or between Lithium-ion batteries and capacitors (*p* > 0.99). Each analytical replicate represented a composite of multiple items within a component category that were homogenized, extracted, and analyzed together; thus, replicates corresponded to independent processing batches rather than individual devices.

**Figure 1:**
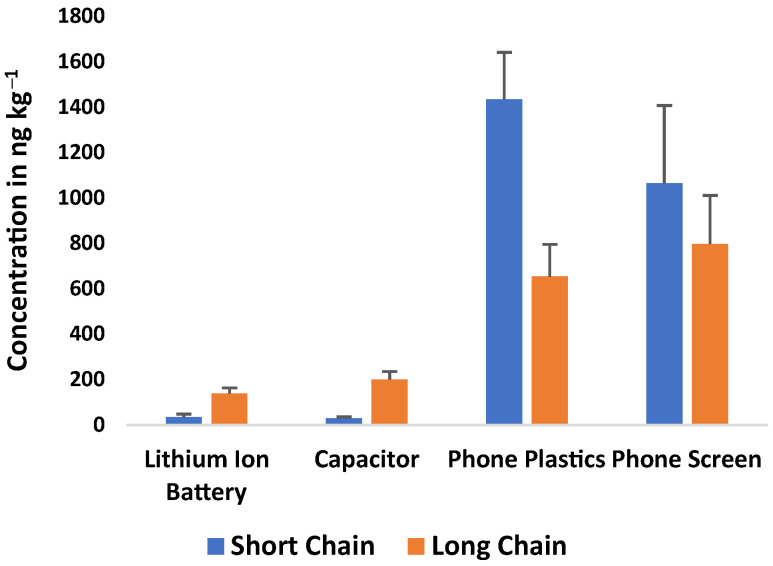
Distribution of short- and long-chain PFAS across e-waste components (number of samples n = 2 independent composite replicates, each comprising ≥4 items).

In Figure 1 and Figure S1, component-specific profiles from our previous study [3] and the findings from this study show similar patterns in short- and long-chain PFAS across the e-waste components. In the previous study [3], short-chain PFAS generally dominated in the e-board (121 ± 16.5 ng·kg^−1^) and cable wire (345 ± 47.5 ng·kg^−1^), whereas the keyboard (205 ± 24.5 ng·kg^−1^) and monitor (27.1 ± 6.56 ng·kg^−1^) exhibited higher contributions from long-chain PFAS. In the current work, phone screens and phone plastics displayed the highest overall PFAS burdens, with short-chain PFAS at higher concentrations than long-chain analogs in both materials. By contrast, Lithium-ion batteries and capacitors were comparatively more enriched in long-chain PFAS despite having lower total PFAS concentrations than the phone screen and plastics. Across all e-waste components, leachable PFAS were dominated by perfluoroalkyl carboxylic acids (PFCAs) and perfluoroalkyl sulfonic acids (PFSAs), with only minor, component-specific contributions from fluorotelomer sulfonic acid (FTSA), fluorotelomer carboxylic acid (FTCA), perfluorooctane sulfonamide (FOSA), perfluoroether carboxylic acid (PFECA), and perfluorooctane sulfonamidoacetic acid (FOSAA), indicating broadly similar class profiles but differing uses of precursors and functionalized PFAS across materials (Figure S2). These results indicate that short-chain PFAS are predominant in plastic and display components, whereas long-chain PFAS are predominant in selected electronic and energy storage components, reflecting different PFAS uses across e-waste components. A similar pattern was observed in our previous study on some e-waste materials [3] (Figure S2).

### 2.2. Phone Screen and Phone Plastics

Phone screens, among other components, contained more of PFCAs, dominated by PFBA (873 ng·kg^−1^), PFHxA (112 ng·kg^−1^), and legacy long-chain PFAS, including PFOA (560 ng·kg^−1^), PFNA (128 ng·kg^−1^), and PFOS (74 ng·kg^−1^). This concurrent presence of highly mobile short-chain acids and more hydrophobic legacy compounds suggests that phone screen assemblies constitute a mixed application that uses legacy fluorochemistries rather than a complete transition away from long-chain PFAS. Similarly, phone plastics exhibited a broad burden, with a ∑PFAS of comparable magnitude but a unique compositional profile. In addition to PFBA (426 ng·kg^−1^), PFHxA (140 ng·kg^−1^), PFPeA (165 ng·kg^−1^), and long-chain acids such as PFNA (206 ng·kg^−1^) and PFOA (178.4 ng·kg^−1^), it contained elevated PFBS (621 ng·kg^−1^) and a small amount of PFDoA (43 ng·kg^−1^), fluorotelomer sulfonates 6:2 FTS (18.7 ng·kg^−1^) and 8:2 FTS (10.5 ng·kg^−1^). This highlights side-chain fluorinated polymers or telomer-based additives, commonly used in formulations designed to provide oil- and water-repellent, flame-retardant, and dielectric properties in polymer housings.

These results align with the trends reported by [3] across multiple e-waste components, where cable wires contained PFBA (136 ng·kg^−1^) and PFHxA (91 ng·kg^−1^) and keyboards had PFOS (70.9 ng·kg^−1^) and PFOA (47.8 ng·kg^−1^). However, fluorotelomer sulfonates in the other components examined (cable wire, keyboard, monitor, and e-board) occurred at lower concentrations than those measured here in phone screens and phone plastics. The comparatively elevated levels in handset components suggest that mobile phones, particularly the screens and polymer housing, constitute PFAS-rich applications within the broader e-waste stream.

Consumer electronics rely on fluorinated coatings for oil- and water-repellency and abrasion resistance. Short-chain PFCAs (e.g., PFBA, PFHxA) and fluoropolymers (historically made with processing aids like PFOA) are widely linked to coatings, paints, and varnishes, including hard-surface and anti-smudge applications [20]. PFNA is a likely candidate for these types of coatings. It has a nine-carbon chain, similar to PFOA, and is known for its non-stick and grease-resistant properties [21]. PFOA has been used as an emulsifier/processing aid in fluoropolymer manufacture, which explains its recurring association with coated glass and polymeric layers in displays [22,23]. Continued reliance on fluorinated repellents for touchscreen oleophobic finishes has been noted even as alternatives emerge [24].

The combination of short-chain PFAAs and alkyl-sulfonates in phone plastics is expected with fluorinated additives or surface treatments used in polymer parts [25]. Short-chain sulfonates (PFBS/L-PFBS) and PFCAs (PFBA/PFPeA/PFHxA) may be used as additives and surfactants to provide mold-release, antistatic, or stain-repellent performance in polymer systems; PFNA has also been associated with plastic processing and fluoropolymer production [24]. These applications might account for the high levels of L-PFBS and PFBA in plastic parts, as well as the broader distribution of shorter-chain acids in polymer matrices. In summary, PFAS in smartphone screens are mainly used as ultra-thin oleophobic and protective surface coatings (and some specialized haptic layers) to provide fingerprint resistance, durability, and smooth touch performance [26].

### 2.3. Capacitors and Lithium-Ion Batteries

**Figure 2:**
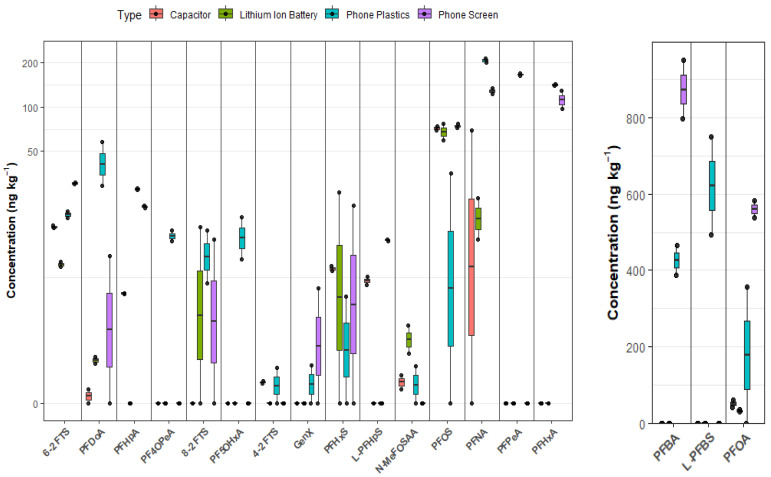
Individual concentrations of PFAS in e-waste components.

As shown in Figure 2, capacitors had a mid-range of ∑PFAS (147–243 ng·kg^−1^) driven by PFOS (71.7 ng·kg^−1^), PFOA (51.6 ng·kg^−1^), PFNA (34.7 ng·kg^−1^), 6:2 FTS (15.5 ng·kg^−1^), and PFHxS/PFHpS (8.0 and 6.57 ng·kg^−1^, respectively). This pattern indicates a legacy signature dominated by sulfonates and C8–C9 carboxylates. While in Lithium-ion batteries, the ∑PFAS (148–158 ng·kg^−1^) is driven by PFOS (68 ng·kg^−1^), PFOA (33 ng·kg^−1^), PFNA (18 ng·kg^−1^), PFHxS (13 ng·kg^−1^), and 6:2 FTS (8.5 ng·kg^−1^). This suggests minor but widespread PFAS use in capacitors and as dielectric films, where PFAS can provide low surface energy, thermal stability, and dielectric properties [26]. This agrees with [19], who reported fluorinated carboxylic acids, including TFAs and PFCAs spanning C3–C8 in selected cells. They suggested that some of these compounds may arise from aging-related degradation of fluorinated battery materials (e.g., PVDF binders or other fluorinated additives) and may reflect differences in cell chemistry and formulation. In Lithium-ion batteries, PFAS are used mainly as polyvinylidene fluoride (PVDF) binders, fluorinated separator/coating components, and occasionally as special additives to provide chemical/thermal stability, adhesion, and wettability in the cell environment [26]. Fluorotelomer sulfonates (e.g., 6:2 FTS) and perfluoroalkyl sulfonates (e.g., PFHxS, PFOS) are widely used as fluorosurfactants and may be added at low concentrations to reduce surface tension and improve the wetting of electrodes and separators in electrochemical systems [24]. In addition, PFOA has been specifically reported as an electrolyte wetting additive that enhances cell performance at very low doses [27]. The EPA’s technical fact sheet also lists PFAS as industrial surfactants, wetting agents, and coatings relevant to electrical applications (e.g., cable wire, casings, and chemical-resistant tubing) [28]. Compared with phone screens/plastics, Lithium-ion batteries do not rely on bulk PFAS mass for function; instead, PFAS appear as binders, special salts, and trace surfactants, accounting for low-level detections. Emerging literature increasingly identifies Lithium-ion batteries as a nexus of PFAS use and emphasizes trace releases during manufacture and recycling [29].

### 2.4. Environmental Burden and Exposure Risks

#### 2.4.1. Concentration vs. Mass Load

Concentration refers to the amount of PFAS per unit weight of a specific component. Mass load refers to the total volume and weight of a component discarded, determining the total quantity of PFAS that enters landfills or recycling operations. For capacitors and Lithium-ion batteries, the ∑PFAS concentrations detected were about 150–240 ng·kg^−1^ due to trace uses such as specialty additives and binders. This makes Lithium-ion batteries a high-hazard material due to the dominance and toxicity of PFOS and PFOA. The primary risk is the widespread presence in ubiquitous devices (phones, tablets, and laptops) and the difficulty of substitution, which ensures a continuous, low-level release into the waste stream [30]. Phone screens: PFAS are applied as a coating on the glass surface. The concentration within the coating layer is very high, often measured in parts per million (ppm), which translates to a much higher ∑PFAS per kg of component material compared to the trace amounts used in batteries/capacitors. The risk implication is twofold: (1) direct human exposure (finger/hand contact as the coating wears) and (2) high leachability (i.e., dissolution or wear of the surface coating easily in water). Phone plastics: Plastic casings, circuit boards, and wiring often incorporate PFAS for flame retardancy, chemical resistance, and insulation, which is a bulk, high-mass application [28]. This component represents the highest environmental load of PFAS in e-waste. Since plastic is the largest mass component of e-waste (17 billion kg globally in 2022) [31], even if the concentration is slightly lower than a pure coating, the total mass of PFAS released from plastics is the most significant environmental threat due to the high tonnage used and disposed. While capacitors and Lithium-ion batteries present a risk characterized by essential, difficult-to-substitute PFAS (with a high hazard but low-level concentration), phone screens, and especially phone plastics, pose a greater risk per mass due to the sheer volume of material and the higher overall ∑PFAS concentration used for bulk functional properties such as coatings and flame retardancy.

#### 2.4.2. Exposure Routes

Risk assessment generally combines three main elements: concentration of the compound, hazard level, and exposure risk. Higher concentrations usually indicate a greater potential hazard. While the detected concentrations of PFAS are very low, they can be significant due to the persistence of PFAS, PFOA, and PFOS, which are carcinogenic. Exposure risk due to direct use during normal phone use is usually low, because PFAS are embedded in materials. However, exposure risks during disposal/recycling can be high, because PFAS can leach into soil/water or volatilize.

The primary exposure route of PFAS associated with e-waste is by leaching and release into the environment at the end-of-life stage during e-waste recycling or disposal operations. PFAS can leach from plastic and screen materials into the soil, groundwater, and air during landfilling or improper dismantling/incineration, posing a higher risk to recycling workers, adjacent communities, and the wider environment. The secondary exposure via direct contact during normal phone use (dermal contact with the screen/casing) would be low because PFAS are typically embedded in the polymer matrix. However, degradation or wear and tear could allow for the release of small particles, especially through hand-to-mouth transfer, which is a concern for children who often handle and mouth objects.

Traditional models often rely on broad categories such as likelihood and consequence, which can oversimplify complex scenarios. The use of weighted factors ensures that the most critical determinants of risk (i.e., hazard severity and exposure likelihood) are prioritized. The risk scoring methodology offers significant improvements over traditional qualitative and semi-quantitative approaches, particularly in its ability to enhance risk ranking and prioritization. This approach decomposes risk into three distinct components: hazard, exposure (with release modifier), and concentration. The release modifier is a dimensionless factor (ranging from 0.1 to 1.0) that adjusts the baseline exposure score to reflect the effectiveness of containment and control measures in reducing the likelihood or magnitude of a hazardous substance being released. It is derived by evaluating engineering controls (e.g., sealed systems, local exhaust ventilation), administrative controls (e.g., handling procedures, restricted access), and environmental conditions that influence release potential [32,33]. For example, a fully enclosed system with verified integrity would receive a low modifier (0.1), while open handling with minimal controls would be assigned a value of 1.0. This approach ensures that the risk score accounts for actual operational conditions rather than theoretical maximum exposure, improving accuracy and prioritization in risk ranking [33,34]).

Risk scores were calculated quantitatively using the following equation:

$$\mathrm{Risk}\;\mathrm{Score}=0.4\;\left(\mathrm{Hazard}\right)+0.4\left(\mathrm{Exposure}\times \mathrm{Release}\;\mathrm{Modifier}\right)+0.2\;(\mathrm{Concentration})$$

This multi-factor approach provides a more nuanced assessment, enabling differentiation between risks that may appear similar under traditional frameworks. This approach is particularly well-suited for regulatory and compliance reporting, as it provides a transparent, quantitative, and reproducible methodology for risk assessment. By using clearly defined components (i.e., hazard, exposure (with release modifier), and concentration) along with explicit weights, the model ensures consistency and objectivity in risk ranking. This structured approach aligns with regulatory expectations for evidence-based decision-making and facilitates clear documentation of how risk scores are derived. The numeric output supports easy integration into compliance dashboards and reporting systems, enabling regulators and stakeholders to verify and audit risk prioritization.

Component-specific release modifiers for the exposure scores were assigned based on how easily PFAS can be released during e-waste processing (e.g., dust generation, leaching, and thermal degradation) as follows:

Phone screen: 0.85 (laminated glass, lower mechanical dust/leach release).

Phone plastic: 0.95 (polymer matrix retention; moderate).

Capacitor: 1.15 (shredding/thermal steps; higher release).

Lithium-ion battery: 1.20 (thermal/chemical processes; highest release).

A quantile-based normalization was used to scale concentration across a common ordinal range (1 = lower-quantile, 2 = middle-quantile, and 3 = upper-quantile). Quantile methods are widely used when variables differ in scale or distribution, as they remove distributional/scale effects, preserve rank information, and are robust to skew typical of environmental concentration data. This approach is standard in other settings (e.g., microarray/toxicity omics, where quantile normalization is applied to place measurements on a comparable distribution without imposing strong parametric assumptions) [35]. In semi-quantitative risk settings, ordinal scoring based on predefined bins (quantiles or bands) is often used to ensure transparency, repeatability, and comparability across heterogeneous inputs [32,34]. The hazard, exposure weights, and concentrations were normalized, and the following qualitative thresholds were used for assigning overall qualitative risk scores:

Low: 1.0–1.6.

Medium: >1.6–2.2.

Medium-High: >2.2–2.7.

High: ≥2.7.

For example, the risk score for PFOA in a phone screens was calculated as follows:

$$\mathrm{Risk}\;\mathrm{Score}\;(\mathrm{Phone}\;\mathrm{screen}-\mathrm{PFOA})=0.4\left(3\right)+0.4\left(2.50\times 0.85\right)+0.2(3)\;=\;2.65$$

Therefore, the quantitative risk score of 2.65 corresponds to the medium-high risk category.

Table 2 compares the relative exposure risks of the e-waste components studied.

### 2.5. Screening of PFAS in E-Waste Samples Using Compound Discoverer v. 3.3

According to the criteria described in Section 3.6, a total of 183 features were filtered out from the e-waste components using Compound Discover and were annotated with the compound name, chemical formula, mass-to-charge ratio (*m*/*z*), retention time (RT), class score, MS^2^ spectra, and matches against the U.S. EPA PFAS master list. The processed dataset is provided in Supplementary Information, Table S2 (Excel). Across the individual e-waste components, including e-board, phone screen, Lithium-ion battery, cable wire, phone plastics, capacitor monitor, and keyboard, a total of 33, 31, 30, 24, 23, 19, 13, and 8 compounds were filtered and tentatively identified as PFAS-related, respectively. These compounds were assigned to confidence levels based on the available evidence [36].

**Figure 3:**
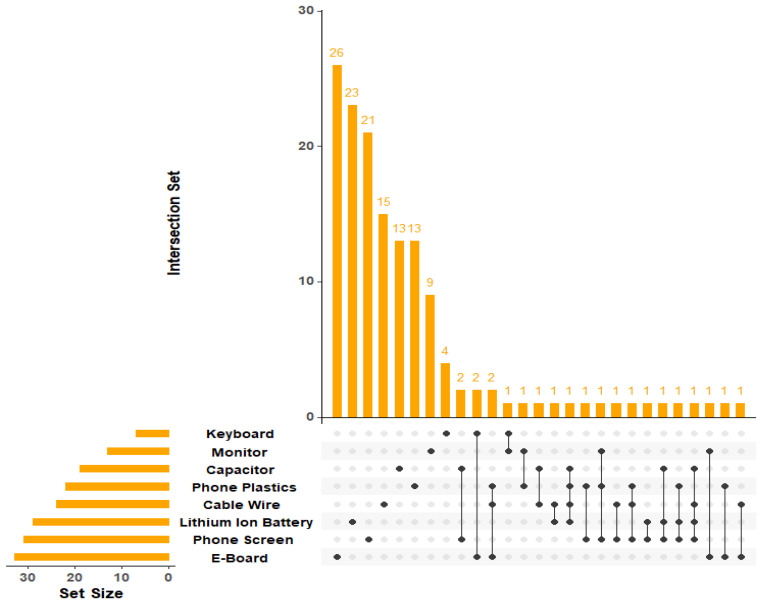
UpSet plot showing shared and unique PFAS features and intersection sizes among e-waste components. Dots indicate the e-waste component in which the detected features were found; values above each bar represent the number of features detected.

The UpSet analysis of PFAS formulas across e-waste components revealed pronounced differences in the number of features tentatively identified and in common with each other in the samples (Figure 3). Component-specific PFAS fingerprints showed a decreasing number of unique formulas in the following order: e-board > phone screen > Lithium-ion battery > cable wire > phone plastics > capacitors > monitor > keyboard. These findings highlight the wide range of PFAS used in complex assemblies such as printed circuit boards and display stacks, where fluorinated chemistries are used in coatings, dielectrics, lubricants, and processing aids, compared with their more limited use in simpler components. The highest intersection bars in the UpSet (Figure 3) plot are dominated by single components, indicating that most tentatively identified PFAS are exclusive to individual materials and that each component exhibits a unique PFAS fingerprint. This component specificity reflects broader evidence that PFAS encode information about product function and manufacturing processes. Only a small subset of formulas appear in multi-component intersections, typically spanning two or three components and comprising no more than four compounds per combination. These shared features likely reflect generic processing aids or legacy additives that occur across multiple applications. Collectively, this suggests that PFAS usage in electronics is highly function- and component-specific, reinforcing the need for targeted management strategies that prioritize PFAS-intensive assemblies rather than treating all e-waste fractions as chemically equivalent.

PFOS, PFOA, and PFBA were confirmed using analytical standards (Confidence Level 1) based on retention time and MS/MS spectral signatures. All other compounds discussed in this section were tentatively identified (Confidence Level 3) based on evidence such as accurate mass and characteristic fragment ions. The full list and confidence levels are provided in Supplementary Table S2. Notably, the most prominent tentatively identified features were associated with well-known PFAS historically used in electronics manufacturing, 3,3,4,4-Tetrafluoro-1,2-dipropylcyclobutene (C_10_H_14_F_4_) and perfluoro-1-octanesulfonic acid (PFOS, C_8_HF_17_O_3_S), which were detected across several core components (cable wire, capacitor, Lithium-ion battery, phone screen, and phone plastics), consistent with the widespread use of fluorinated surfactants and processing aids in circuit boards, wiring insulation, coatings, and assembly processes reported in previous studies [3]. In addition, the overlap of perfluorobutanesulfonic acid (PFBS, C_4_HF_9_O_3_S) between the monitor, phone plastics, and the phone screen and the occurrence of perfluorooctanoic acid (PFOA, C_8_HF_15_O_2_) in cable wire, phone plastics, and the phone screen aligns with the literature indicating that PFBS is widely used as a short-chain replacement in fluoropolymer production and surface-treatment formulations, whereas PFOA represents a legacy processing aid historically employed in fluoropolymer manufacture and polymer processing. Besides sulfonic and carboxylic PFAS, the non-targeted analysis identified several fluorinated compounds with strong plausible links in electronic materials and processing chemistries. In phone plastics, 1H-perfluorohexane (C_6_ H_13_ F) was among the most intense fluorinated features. Product descriptions and regulatory assessments identify 1H-perfluorohexane as a highly stable hydrofluorocarbon used as a carrier fluid for precision cleaning of sensitive electronic components and as a heat-transfer fluid in the electronics industry, underscoring its relevance to electronic assemblies [37]. Cyclic fluorocarbon 3,3,4,4-tetrafluoro-1,2-dipropylcyclobutene (C_10_H_14_F_4_) was also identified in the phone screen and plastics. The compound appears on electronics sector “banned and restricted substances” lists (Qorvo’s PFAS inventory) as a substance prohibited from use in products, indicating that it is associated with electronic chemical supply chains to warrant explicit restriction (Qorvo 2025) [38]. In the Lithium-ion battery, ethyl trifluoroacetate (C_4_H_5_F_3_O_2_) was among the most intense features. It is widely marketed as a fluorinated intermediate and solvent for liquid-crystal and other electronic-chemical applications and is explicitly listed by suppliers under “electronic chemicals/for OLED,” as well as in patents describing its use in the synthesis of fluorinated liquid-crystal and photoactive materials [39].

Most notably, our NTA screening revealed two higher-mass PFAS that recur across multiple device fractions, 3,3,4,4,5,5,6,6,7,7,8,8,9,10,10,10-hexadecafluoro-9-(trifluoromethyl)decan-1-ol (1H,1H,2H,2H-perfluoro-9-methyldecan-1-ol) (C_11_H_5_F_19_O) and dimethyl (3,3,4,4,5,5,6,6,7,7,8,8,9,9,10,10,11,11,12,12,12-henicosafluorododecyl) phosphonate (C_14_H_10_F_21_O_3_P), in consumer electronic components. Fluorotelomer alcohol was detected in both the keyboard and monitor, whereas the perfluoroalkyl phosphonate occurred in the Lithium-ion battery and phone screen. Both compounds are commercially available fluorinated intermediates/surfactants: 1H,1H,2H,2H-perfluoro-9-methyldecan-1-ol (9Me 8:2 FTOH) is listed in PFAS catalogs used to characterize electronics-related PFAS sources and appears among fluorotelomer alcohols associated with PFAS emissions from electronics fabrication and wastewaters; meanwhile, dimethyl (1H,1H,2H,2H-perfluorododecyl)phosphonate (the corresponding C_12_ perfluoroalkyl phosphonate) is sold as a highly fluorinated phosphonate and grouped with other perfluoroalkyl phosphonic/phosphinic acids that are used as commercial fluorinated surfactants and surface-treatment agents (leveling and wetting agents) in industrial coatings [40,41]. Both chemistries have been described as building blocks for fluorinated polymers, surface-treatment agents, and high-performance coatings, including applications relevant to advanced polymeric and electronic materials. However, previous investigations of PFAS in e-waste have generally focused on perfluoroalkyl acids, fluorotelomer sulfonates, and a limited set of fluorotelomer alcohols, and have not explicitly identified these specific structures in device fractions. Their occurrence across multiple components suggests that fluorotelomer-based PFAS and perfluoroalkyl phosphonates are more broadly integrated into electronic assemblies than currently documented. This finding highlights the value of NTA for revealing previously unreported PFAS chemistries in e-waste and indicates that surfactants and surface-modifying agents used in coatings, photoresists, and wet-processing steps may represent additional, under-recognized PFAS source terms in electronic products.

**Figure 4:**
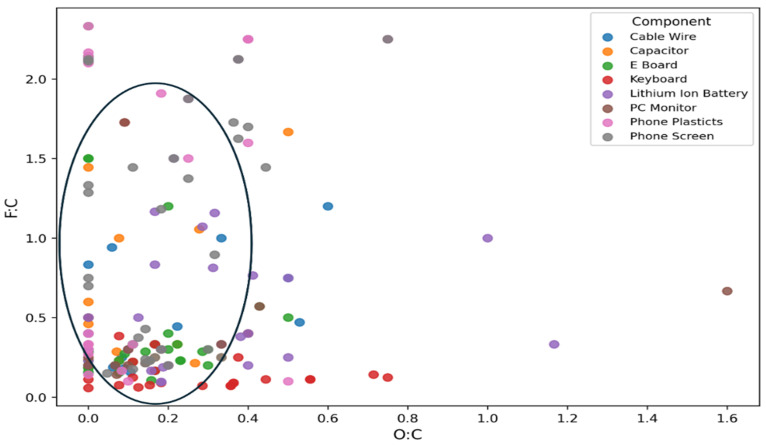
Van Krevelen diagram of PFAS screened from leachate of different e-waste components using CD v. 3.3. The circle indicates the more densely populated area in the region, with F:C from 0.25 to 2.0 and O:C from 0 to 0.4, suggesting that the majority of the PFAS have a low oxygen content and are polyfluoroalkyls.

A Van Krevelen plot, as shown in (Figure 4), is also presented to visualize the detected PFAS features of the e-waste samples from different sources in which the ratio of fluorine and carbon (F:C) is plotted against the ratio of oxygen to carbon (O:C) for each feature. Based on the degree of saturation and oxygen content, the features are localized in different regions on the Van Krevelen diagrams.

As described in other studies, the compounds containing no oxygen (aromatic hydrocarbons) appear at the y-axis [42]. PFAS with a high content of fluorine would appear in the upper region, given that the F:C ratio is relatively high, especially for the legacy perfluorinated compounds, where all the hydrogens on the carbon chain are substituted by fluorine [43]. The clutter of more densely populated areas appears to be in the region with F:C from 0.25 to 2.0 and O:C from 0.0 to 0.4, which suggests the majority of the PFAS have a low oxygen content and are likely polyfluoroalkyls, where H atoms are only partially substituted by fluorine in the carbon chain [44].

**Figure 5:**
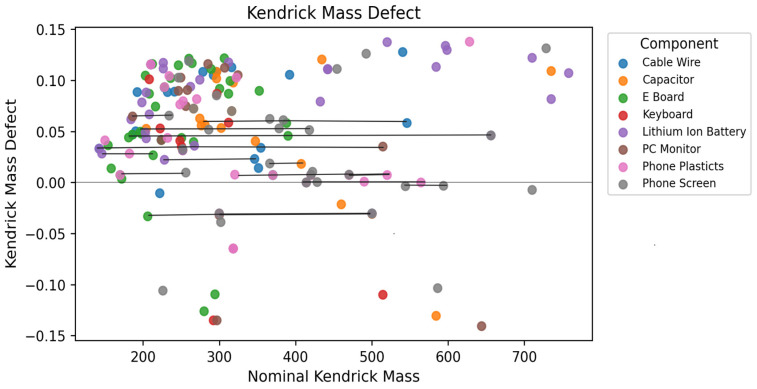
Kendrick mass defect (KMD) plot of PFAS features detected in leachates from eight electronic waste components. The horizontal bands of points with similar KMD values and increasing nominal Kendrick mass indicate CF_2_-based homologous series annotated with horizontal connecting lines, while the distribution of points across components highlights both shared and component-specific PFAS signatures.

The Kendrick mass defect (KMD) plot was used to visualize the CF_2_-based homologous series among the PFAS-like features detected in the eight electronic waste components. In the KMD plot, most features clustered between nominal Kendrick masses of 150–350 with positive KMD values (0.03–0.12). The horizontal bands indicate multiple CF_2_-homologous series, consistent with families of perfluoroalkyl substances differing mainly by successive CF_2_ units [45]. The substantial overlap of features from several components within these bands (Figure 5) suggests that PFAS formulations or additives are used across multiple e-waste materials.

At higher nominal Kendrick masses (400–750), fewer but more widely dispersed PFAS-like features were observed, spanning both positive and negative KMD values. Similar dispersive patterns have been reported for partially fluorinated and structurally complex PFAS classes, which deviate from the tight CF_2_-based homologous series typical of perfluoroalkyl acids. Therefore, this region likely contains polyfluoroalkyl phosphates, sulfonamide-based PFAS, and other oligomeric additives in which CF_2_ repetition is less dominant. High-mass features were particularly associated with phone screens, Lithium-ion batteries, and selected plastic components, suggesting that PFAS chemistries are more specific to display coatings, electrolytes, or polymeric binders. Features with KMD values close to 0.0 are likely to correspond to highly perfluorinated chains, while those with larger positive or negative KMD values may contain additional heteroatoms or mixed aromatic/aliphatic backbones. In summary, KMD analysis confirms that the non-target features form distinct CF_2_-based homologous series and reveals both widely shared and component-specific PFAS fingerprints within the e-waste matrix [44,46].

## 3. Materials and Methods

### 3.1. Chemicals and Materials

All chemicals and reagents used in this research were LC–MS grade and purchased from Fisher Scientific (Hampton, NH, USA). Native standards, isotopically labeled surrogates, and internal standards were prepared in methanol from certified reference stocks supplied by Wellington Laboratories (Guelph, ON, Canada). Consumables, including centrifuge tubes, disposable pipette tips, and vial caps, were made of polypropylene (PP) and bought from Fisher Scientific. Additional details on chemicals and materials are provided in the Supplementary Information (Section S3.1 and Table S1).

### 3.2. E-Waste Sampling and Sample Processing

**Figure 6:**
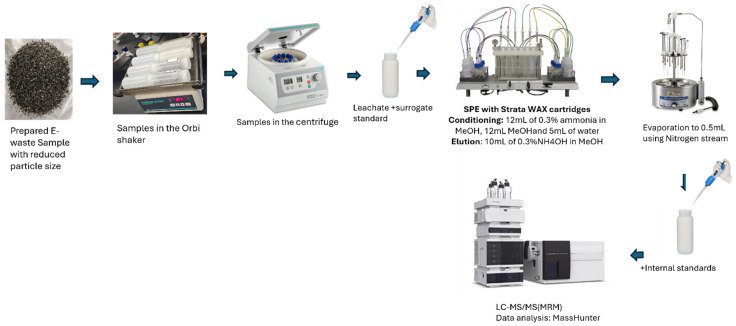
PFAS sample preparation, leaching, extraction, and analysis workflow.

E-waste components, including phone screens, capacitors, Lithium-ion batteries, and phone plastics, were collected at random from the Steven T. Smith (STS) electronic waste recycling facility in Miami, FL, USA. To process the samples before leaching experiments, the materials were disassembled, separated, sorted, and shredded (the Lithium-ion battery was carefully and gently disassembled in a closed space to avoid ignition). The phone screen and plastic casing samples were cut and shredded using a stainless-steel blender, then sieved to obtain particles between 2 mm and 6 mm. Lithium-ion batteries were cut to 5 mm. The experiments maintained this particle size to ensure consistency across all tested materials. Each type of e-waste component was processed as a composite sample. Composite samples were prepared by combining over 4 representative items, homogenizing, and extracting them as a single analytical sample. Two independent composite replicates (*n* = 2) were prepared and analyzed for each component type, with each replicate representing a separate processing batch rather than an individual device. A schematic workflow of sample processing, leaching, extraction, and analysis is provided in Figure 6.

### 3.3. Leaching Experiments

For the leaching experiments, samples were broken and sieved (2 and 6 mm). Duplicate tests were conducted for each composite sample. Thirty grams of each sample was placed into a 500 mL polypropylene (PP) bottle, followed by the addition of 300 mL of ultrapure PFAS-free deionized water (DIW) (LabChem HPLC grade). Ultrapure (HPLC-grade) DIW was employed to avoid contributions from extraneous cations and anions that could be present with other contaminants. Deionized (DI) water was used to minimize matrix interference and ensure that measurements in leachate originate from the e-waste samples only. Leaching experiments were performed at a solid-to-liquid ratio of 1:10, consistent with ratios commonly used in standardized leaching protocols (e.g., the U.S. EPA Leaching Environmental Assessment Framework, LEAF) [47]. Given the low detection limits achievable for PFAS, a 1:10 ratio is appropriate for PFAS leaching studies, and this same methodology has been used in our previous study [3]. The samples were placed on an orbital shaker and agitated at 100 rpm for 30 days at room temperature.

After the agitation period of 30 days, the samples were centrifuged at 4500 rpm (Sorvall ST 8, Thermo Fisher Scientific, Waltham, MA, USA) for 30 min. The supernatant was carefully transferred into a separate, clean 500 mL PP bottle and stored in the refrigerator (4 °C) for subsequent PFAS extraction and analysis.

### 3.4. PFAS Sample Extraction and Analysis

A 300 mL aliquot of leachate from each e-waste component, along with associated procedural blanks and blank-spiked samples, was subjected to solid-phase extraction (SPE) using Strata-XL AW weak anionic exchange cartridges (Phenomenex, Torrance, CA, USA) on semi-automated SPE equipment for the extraction and preconcentration of PFAS, followed by liquid chromatography–tandem mass spectrometry (LC-MS/MS). The resulting extracts were injected into an Agilent 1290 Infinity II LC system coupled to an Agilent 6470 triple-quadrupole mass spectrometer (Agilent Technologies, Santa Clara, CA, USA) equipped with an Agilent Jet Stream electrospray ionization (AJS-ESI) source (Figure 6). The method quantitatively targeted 40 PFAS, including both legacy and emerging compounds (Table S1), consistent with analytes listed in U.S. EPA Method 1633. To ensure data quality, routine batch quality assurance and control (QA/QC) procedures were implemented, including analysis of laboratory blanks, laboratory-fortified blanks, triplicate samples, and a calibration verification standard prepared by an independent source. A detailed description of the SPE procedure, chromatographic and mass spectrometric operating conditions, method performance metrics, and QA/QC outcomes is provided in the Supporting Information (Section S3.4 and Tables S3–S6). In accordance with U.S. EPA Method 1633, two MRM transitions were monitored for each PFAS whenever feasible, one for quantitation and one for confirmation. However, certain short-chain PFAS (e.g., PFBA, PFPeA) exhibit limited fragmentation, producing only a single reliable product ion under optimized conditions. In these cases, identity was confirmed using retention time agreement with calibration standards, isotope-dilution internal standards, and all required quality control checks, consistent with Method 1633 recommendations.

### 3.5. Data Processing for Targeted Analysis

Targeted PFAS quantification was limited to four matrices (capacitor, Lithium-ion battery, phone plastics, and phone screen) because targeted data for cable wire, keyboard, e-board, and monitor were reported previously [3]; however, all eight components were included in the NTA to enable comprehensive screening for additional and potentially unreported PFAS features across e-waste matrices. Quantitative analysis of PFAS was performed using MassHunter QQQ Quantitative analysis software version 10.0, applying decision rules consistent with established protocols [48,49]. Chromatographic peaks were accepted only when they satisfied all of the following criteria: (i) retention time agreement within ±0.1 min of the corresponding internal standard or native calibration standard, (ii) presence of a confirmation transition where available, (iii) a signal-to-noise ratio greater than 3, and (iv) measured concentrations exceeding the method detection limit (MDL). Results not meeting these requirements were reported as <MDL, and values below the MDL were excluded from the computation of ΣPFAS. Final analyte levels were converted to ng/kg using the calculation outlined below:

$$\mathrm{PFAS}\;\mathrm{concentrations}\;({\mathrm{ng}\cdot \mathrm{kg}}^{-1})\;=\;\frac{\mathrm{PPFAS}\;\mathrm{Concentration}\;\mathrm{in}\;\mathrm{leachate}\left(\frac{ng}{L}\right)\times 1000\;g/kg)}{We(g)/Vl(L)}$$

where We is the weight of the e-waste component (grams), and Vl is the volume of DI water used in the leaching experiment (liters).

### 3.6. PFAS Screening for Non-Targeted Analysis Identification

For PFAS screening, an ultra high-performance liquid chromatography (UHPLC) system was coupled to an Orbitrap^TM^ Astral^TM^ hybrid high-resolution mass spectrometer (Thermo Scientific, Waltham, MA, USA) operated in negative electrospray ionization. The full scan has a range of *m*/*z* 100–800 and a resolution of 120,000 FWHM and >80,000 FWHM for the MS2 scan. The data-dependent acquisition (DDA) mode was used to acquire MS2 data. Information about the instrument conditions is shown in Supplementary Tables S7–S9.

### 3.7. NTA Data Processing: Compound Discover^TM^ (CD) v. 3.3

Data processing and annotation were carried out using the Compound Discoverer workflow, “Environmental w Stats Unknown ID w Online and Local Database Searches” integrated in the software (Figure S2). The databases queried included ChemSpider, mzCloud, mzVault, and the PFAS Master List in the U.S. EPA DSSTox. Peak detection parameters included a mass tolerance of 5 ppm and a minimum peak intensity threshold of 50,000. Elemental composition-based molecular formula prediction was conducted using maximum element constraints of C_90_, H_190_, Br_3_, Cl_4_, F_40_, N_10_, O_18_, Na_2_, P_3_, and S_5_, with the hydrogen-to-carbon (H:C) ratio limited to a maximum of 3.5. The mass tolerance was set to <5 ppm with a signal-to-noise ratio (S/N) of 3, and blank subtraction was automatically applied by the software algorithm using a procedural blank. The list of PFAS features initially generated in Compound Discoverer (CD) was further filtered to increase identification confidence. Only features satisfying all of the following conditions were retained for subsequent analysis: (1) mass defect ≥0.85 or ≤0.10, (2) a molecular formula was successfully assigned, and (3) an MS^2^ spectrum was acquired for the preferred ion in data-dependent acquisition (DDA). The identified chemicals were assigned confidence levels ranging from 1 to 4 based on established rules [36].

## 4. Conclusions

This study demonstrates that electronic waste components are a relevant and under-recognized source of leachable PFAS, with marked differences in both concentrations and chemical fingerprints across device fractions. Phone screens and phone plastics consistently exhibited the highest ∑PFAS in leachates, dominated by short-chain PFAAs (PFBA, PFHxA) and legacy acids (PFOA, PFNA), whereas capacitors and Lithium-ion batteries contained lower overall PFAS loads but showed mixed sulfonate/carboxylate signatures (PFOS, PFHxS, and 6:2 FTS) consistent with dielectric and wetting applications. While the concentrations of total PFAS in internal components such as capacitors and Lithium-ion batteries appear low (approximately 150–240 ng/kg), their inclusion as critical additives (e.g., binders and wetting agents) makes their substitution difficult.

Non-targeted analysis revealed PFAS features forming CF_2_-based homologous series with predominantly per- and polyfluoroalkyl characteristics and low oxygen content. The predominance of component-specific features, combined with a smaller set of shared compounds (e.g., PFOS, PFOA, fluorotelomer alcohols, and phosphonates), indicates that both unique formulations and common manufacturing steps govern PFAS fingerprints across circuit boards, wiring, displays, plastics, and electrochemical components. These results confirm that targeted lists alone capture only a fraction of the fluorinated chemical space present in e-waste, highlighting the need for integrated targeted/NTA workflows to identify emerging and proprietary PFAS in complex solid waste matrices.

From a management and policy perspective, our findings support the inclusion of PFAS considerations in e-waste regulations, product design, and end-of-life infrastructure. Prioritizing high-leachability fractions such as phone screens and plastic housings in collection, treatment, and disposal strategies could reduce PFAS releases, while upstream measures, such as substituting non-fluorinated coatings where feasible, phasing out high-hazard legacy PFAS, and improving the disclosure of fluorinated additives, would help mitigate future burdens. At the same time, recognizing Lithium-ion batteries and capacitors as a nexus of specialized but persistent PFAS uses is critical for designing safer battery chemistries and recycling systems as the energy storage sector expands.

This work is subject to several limitations, including the use of deionized water in a single leaching scenario, a finite set of targeted PFAS, and reliance on tentative identifications for many non-targeted features. Future studies should examine PFAS leaching under a broader range of environmental conditions (e.g., landfill leachate and varying pH/ionic strength), explore time-dependent leaching kinetics, and further characterize transformation products and the toxicological relevance of newly identified PFAS. Nevertheless, by linking leaching behavior, risk quotients, and high-resolution chemical profiling across multiple e-waste components, this study provides a crucial foundation for understanding and managing electronic waste’s contribution to the global PFAS contamination burden.

## Figures and Tables

**Table 1:** PFAS concentrations in selected e-waste leachates, reported as mean  ±  SD (range).

PFAS	DF (%)	Phone Screen (ng·kg^−1^)	Lithium-Ion Battery (ng·kg^−1^)	Capacitor (ng·kg^−1^)	Phone Plastics (ng·kg^−1^)
PFBA	50.0	873 ± 108	<0.0900	<0.0900	426 ± 54.4
PF4OPeA	25.0	<0.0400	<0.0400	<0.0400	13.4 ± 1.58
PFPeA	25.0	<0.0400	<0.0400	<0.0400	165 ± 4.60
L-PFBS	25.0	<0.0200	<0.0200	<0.0200	621 ± 182
PFHxA	50.0	112 ± 22.3	<0.0200	<0.0200	140 ± 3.44
GenX	25.0	2.88 ± 4.08	<0.0400	<0.0400	0.634 ± 0.896
PFHxS	63.0	10.7 ± 15.2	13.3 ± 18.8	8.00 ± 0.472	2.53 ± 3.58
PFHpA	75.0	21.2 ± 0.480	<0.0200	5.32 ± 0.0800	27.8 ± 0.615
6-2 FTS	100	30.4 ± 0.759	8.53 ± 0.476	15.5 ± 0.520	18.7 ± 1.29
PFHpS	50.0	12.5 ± 0.139	<0.0600	6.57 ± 0.585	<0.0600
PFOA	88.0	560 ± 31.7	33.3 ± 4.29	51.6 ± 12.4	178 ± 252
PFOS	88.0	74.2 ± 3.23	68.0 ± 12.3	71.7 ± 3.60	17.7 ± 25.1
PFNA	88.0	128 ± 7.76	18.4 ± 8.23	34.7 ± 49.0	206 ± 9.20
8-2 FTS	50.0	6.30 ± 8.91	7.70 ± 10.9	<0.0200	10.5 ± 5.96
N-MeFOSAA	63.0	<0.0600	2.37 ± 0.938	0.676 ± 0.324	0.614 ± 0.868
PFDoA	75.0	4.88 ± 6.89	1.44 ± 0.190	0.228 ± 0.322	43.5 ± 20.2
∑PFAS	-	1739–1932	148–158	147–243	1575–2197

Values are reported as mean ± SD for n = 2 independent composite replicates, each comprising ≥4 items. DF = detection frequency. SD = standard deviation.

**Table 2:** Hazard levels and relative exposure risks for PFAS detected in the e-waste components studied.

Component	Compound	Conc. (ng·kg^−1^)	Hazard	Exposure	Release Potential Modifier	Hazard Score	Exposure Score	Adjusted Exposure Score	Concentration Score	Risk Score (Quantitative)	Overall Risk Score (Qualitative)
Phone Screen	PFOA	560	High	Low (Direct Use), High (E-Waste)	0.850	3.00	2.50	2.13	3	2.65	Medium-High
	PFBA	873	Medium	Low (Direct Use), High (E-Waste)	0.850	2.00	2.50	2.13	3	2.25	Medium-High
	PFOS	74.0	High	Low (Direct Use), High (E-Waste)	0.850	3.00	2.50	2.13	3	2.65	Medium-High
Phone Plastic	PFBS	621	Medium-High	Low (Direct Use), High (E-Waste)	0.950	2.50	2.50	2.38	3	2.55	Medium-High
	PFNA	206	High	Low (Direct Use), High (E-Waste)	0.950	3.00	2.50	2.38	3	2.75	High
	6:2 FTS	18.7	Medium	Low (Direct Use), High (E-Waste)	0.950	2.00	2.50	2.38	1	1.95	Medium
Capacitor	PFOS	71.7	High	High	1.15	3.00	3.00	3.45	2	2.98	High
	PFOA	51.6	High	High	1.15	3.00	3.00	3.45	2	2.98	High
	PFNA	34.7	High	Medium-High	1.15	3.00	2.50	2.88	2	2.75	High
	6:2 FTS	15.5	Medium	Medium	1.15	2.00	2.00	2.30	1	1.92	Medium
	PFHxS/PFHpS	8.00	High	Low	1.15	3.00	1.00	1.15	1	1.86	Medium
Lithium-ion Battery	PFOS	68.0	High	High	1.20	3.00	3.00	3.60	2	3.04	High
	PFOA	33.0	High	Medium	1.20	3.00	2.00	2.40	2	2.56	Medium-High
	PFNA	18.0	High	Low-Medium	1.20	3.00	1.50	1.80	1	2.12	Medium
	PFHxS	13.0	High	Low	1.20	3.00	1.00	1.20	1	1.88	Medium
	6:2 FTS	8.50	Medium	Low	1.20	2.00	1.00	1.20	1	1.48	Low

## Data Availability

The data supporting the findings of this study are available in the Supporting Information or from the corresponding author upon reasonable request.

## Supplementary Materials

The following supporting information can be downloaded at: https://www.mdpi.com/article/10.3390/molecules31030445/s1, Table S1. List of 40 PFAS with their internal standards applied for quantitation, list of 24 PFAS in the surrogate standard, and the 22 PFAS mixture used as secondary standards; Table S2. Tentatively identified PFAS features by Compound Discovered v 3.3 in e-waste components; Table S3. Liquid chromatograph (LC) gradient conditions, using (A) 2 mM ammonium acetate, and (B) methanol as the mobile phases; Table S4. Mass spectrometer (MS) parameters for liquid chromatography (LC)-MS/MS analysis; Table S5. Summary of the dynamic Multiple Reaction Monitoring (dMRM) conditions for MS/MS analyses; Table S6. Method performance for detected PFAS in this study; Table S7. Liquid Chromatograph (LC) gradient conditions for the Orbitrap; Table S8. MS parameters for Full MS and data-dependent MS (ddMS2); Table S9. ddMS^2^ Trigger and Exclusion Parameters; Figure S1. Distribution of short- and long-chain PFAS across e-waste components from a previous study; Figure S2. Proportion of PFAS classes in the e-waste leachate; Figure S3. Compound Discoverer workflow for the screening of PFAS.
