# Supplementary material for: Leachability and Chemical Profiles of Per- and Polyfluoroalkyl Substances in Electronic Waste Components: Targeted and Non-Targeted Analysis
Source: Molecules. 2026 Jan 27;31(3):445. doi: 10.3390/molecules31030445 (PMC12898688; doi:10.3390/molecules31030445)
Supplement: Supplementary file 1 [file molecules-31-00445-s001.zip › SI 4058886 e-waste paper revised.pdf]

Leaching Behavior and Chemical PFAS Profile in Electronic-Waste Components by Non-Targeted Analysis (NTA).

Joshua O. Ocheje[1,2], Yelena Katsenovich[3], Berrin Tansel[4], Natalia Quinete[1,2]

[1] Department of Chemistry and Biochemistry, Florida International University, Miami, 33199, FL, US

[2] Institute of Environment, Florida International University, North Miami, FL, 33181, US

[3] Applied Research Center, Florida International University, Miami, FL, 33174, US

[4] Civil and Environmental Engineering Department, Florida International University, Miami, FL, 33174, US

Supplementary Information:

3.1 Chemicals and Materials Description

3.4 PFAS sample extraction and analysis

Table S1. List of 40 PFAS with their internal standards applied for quantitation, list of 24 PFAS in the surrogate standard and the 22 PFAS mixture used as secondary standards.

Table S2. Tentatively identified PFAS features by Compound Discovered v 3.3 in e-waste components. (Excel file)

Table S3. Liquid chromatograph (LC) gradient conditions, using (A) 2mM ammonium acetate, and (B) methanol as the mobile phases.

Table S4. Mass spectrometer (MS) parameters for liquid chromatography (LC)-MS/MS analysis.

Table S5. Summary of the dynamic Multiple Reaction Monitoring (dMRM) conditions for MS/MS analyses.

Table S6. Method performance for detected PFAS in this study.

Table S7. Liquid Chromatograph (LC) gradient conditions for the Orbitrap

Table S8. MS parameters for Full MS and data-dependent MS (ddMS2)

Table S9. ddMS^2 Trigger and Exclusion Parameters

Figure S1. Distribution of short- and long-chain PFAS across e-waste components from a previous study.

Figure S2. Proportion of PFAS classes in the e-waste leachate.

Figure S3. Compound Discovered workflow for the screening of PFAS.

3.1 Chemicals and Materials

A native PFC standard mix containing 25 PFAS (PFAC-MXH, 1000-5000 µg/L), a native replacement PFAC standard mix (PFAC-MXF, 2000 µg/L), a native perfluoroalkyl ether carboxylic acids and sulfonate mixture (PFAC-MXG, 2000 µg/L), a native N-Me/EtFOSA & N-Me/EtFOSE standard mixture (PFAC-MXI, 1000- 10,000 µg/L), a native X:3 fluorotelomer carboxylic acid standard mixture (PFAC-MXJ, 4000-20,000 µg/L), a mass-labeled PFAS extraction standard solution mixture containing 24 labeled PFAS (MPFAC-HIF-ES, 250-2000 µg/L) and a mass-labeled PFAS injection standard solution mixture containing 7 labeled PFAS (MPFAC-HIF-IS, 250-2000 µg/L) were purchased from Wellington Laboratories Inc. (Guelph, Ontario, Canada). A secondary standard containing 24 native PFAS standard (PFC-24, 2000 µg/L in methanol) was bought from AccuStandard (New Haven, CT, USA) and used for initial calibration verification (ICV). Working solutions were prepared by diluting the stock solutions (in methanol) to 1 and 10 µg/L (based on the lowest concentrations present in the standards) in LC-MS grade water, and further stored refrigerated at 4 °C. A list of all native PFAS analyzed, including their full name, abbreviations, molecular formula, and weight, and assigned labeled standards is shown in Table S1. All solvents and reagents used, which included Optima LC-MS grade methanol, water, hexane, acetone, acetonitrile, ammonium hydroxide, and ammonium acetate, were obtained from Fisher Scientific (Waltham, MA, USA). The solid phase cartridges used were Strata-XL-AW 100 µm (500 mg/3 mL) from Phenomenex (Torrance, CA, USA). Laboratory supplies such as centrifuged tubes, disposable pipette tips, vial caps used were made of polypropylene (PP).

3.4 PFAS sample extraction and analysis

To avoid cross-contamination, all containers, bottles, and tubing used during extraction and sample preparation were rinsed twice with solvents of different polarities: hexane, acetone, acetonitrile and methanol, and have been previously tested for potential PFAS contamination. Leachate samples were processed through solid phase extraction (SPE) using Strata-XL AW weak anionic exchange cartridges on a semi-automated SPE equipment for extraction and preconcentration of PFAS. In short, cartridges were successively pre-conditioned with 12 mL of 0.3% ammonium hydroxide in methanol, 12 mL of methanol and equilibrated with 5 mL of water, before loading of 45 mL e-waste leachate sample spiked with 50 µL of the labeled extraction standard (MPFAC-HIF-ES) mixture (2.5 ng mL^{-1}). Samples were loaded into the cartridges under vacuum and after all has passed, cartridges are left to dry for about 1 hour. In the second stage, cartridges were eluted

with 10 mL of 0.3% ammonium hydroxide in methanol, which is further evaporated to dryness under a gentle nitrogen flow in a heated water bath at 40 °C, then reconstituted to a 450 µL volume with 95:5% (vol/vol) 2mM ammonium formate/methanol. For quality control purposes, blanks, spiked blanks, and duplicate analyses were processed for each experiment through the same procedure as the samples. Blank samples consisted of 45 mL LC-MS grade water spiked with the labeled extraction standard (MPFAC-HIF-ES) mixture (2.5 ng mL^{-1}), while spiked blanks were prepared with 45 mL LC-MS grade water spiked with 250 µL of 2.5 ng mL^{-1} of native standard mixture (containing 40 PFAS from PFAC-MXF, PFAC-MXG, PFAC-MXH, PFAC-MXI and PFAC-MXJ) and 50 µL the labeled extraction standard (MPFAC-HIF-ES) mixture (2.5 ng mL^{-1}).

Reconstituted samples are transferred into LC polypropylene (PP) vials, 50 µL of 2.5 ng mL^{-1} labeled internal standard mixture (MPFAC-HIF-IS) were added and samples were kept refrigerated at 4°C until LC-MS/MS analysis. 100 µL of samples were injected and analyzed by an Agilent 1290 Infinity II LC interfaced to an Agilent 6470 triple quadrupole LC-MS/MS system equipped with Agilent Jet Stream electrospray ionization (ESI) source in negative mode. The LC system was modified with PFAS free tubing and a delay column (Hypersil GOLD aQ C18, 20 × 2.1 mm, 12 µm) was placed between the mobile phase mixer and the sample injector. A Hypersil GOLD pentafluorophenyl (PFP) column (150 mm × 2.1 mm, 3 µm) with a PFP guard column (Hypersil Gold PFP 5 µm drop-in guards) was used as analytical column for PFAS separation and maintained at a temperature of 50 °C using 95:5 2mM ammonium acetate:methanol and methanol as mobile phases in a flow rate of 0.4 mL min^{-1}. Sample acquisition was performed using a multiple-reaction monitoring (MRM) method in negative mode for the simultaneous quantification of 40 PFAS, which included when available two transitions per compound for quantitative and identification (qualitative) purposes. Chromatographic conditions, mass spectrometry parameters and monitored transitions are displayed in Tables S2, S3 and S4.

QA/QC measures for sample processing included the use of contaminant-free tubes (polypropylene bottles, container, and vials) and glassware, certified internal standards and verification of PFAS-free solvents and reagents. Laboratory procedural blanks were prepared and measured to monitor the performance of the laboratory procedures and to assure that PFAS were not originally from the laboratory. PFAS found in the e-waste samples were subtracted from the ones identified in the blank. All quantifications were performed by the internal standard approach, which accounts for potential losses during the extraction step, as it is well known to reduce matrix

effects, where sample concentrations will be calculated based on the analyte/isotopically labeled standard area ratios. Typical batch QA/QC procedures are in place to assess not only cleanliness but also precision and accuracy; they include, besides the analysis of laboratory blanks, laboratory fortified blank (LFB), sample duplicates, and the analysis of a calibration verification standard from a different source. A continuous calibration verification (CCV) solution will be injected every 20 or fewer injections, and the measured concentration should not deviate more than 30% from the assigned value. In case of deviation, the instrument was cleaned and recalibrated before proceeding with the following injections. Target compounds in samples were qualitatively identified by comparing retention times (RTs) to RTs of isotopically labeled surrogates in the same sample. QC procedures followed EPA method 1633 to ensure the proper operation of sample preparation and cleanup techniques. These analyses will help to measure PFAS content in e-waste samples and prioritize the most common PFAS homologues for further monitoring.

Table S1. List of 40 PFAS with their internal standards applied for quantitation, list of 24 PFAS in the surrogate standard and the 22 PFAS mixture used as secondary standards.

Abbreviation	Compound Name	Molecular Formula	Molecular Weight	I.S.	Secondary Standard
4-2 FTS	Sodium 1H,1H,2H,2H-perfluoro-1-hexanesulfonate	$C_6H_4F_9O_3S.Na$	350.13	13C2 4-2 FTS	✓
6-2FTS	Sodium 1H,1H,2H,2H-perfluoro-1-octanesulfonate	$C_8H_4F_{13}O_3S.Na$	450.15	13C2 6-2FTS	✓
8-2 FTS	Sodium 1H,1H,2H,2H-perfluoro-1-decanesulfonate	$C_{10}H_4F_{17}O_3S.Na$	550.17	13C2 8-2 FTS	✓
ADONA	Sodium dodecafluoro-3H-4,8-dioxanonanoate	C7HF12O4.Na	400.05	M13C4 PFHpA	
FOSA	Perfluoro-1-octanesulfonamide	$C_8H_2F_{17}NO_2S$	499.14	13C8 FOSA	
HFPO-DA	2,3,3,3-Tetrafluoro-2-(1,1,2,2,3,3,3-heptafluoropropoxy)-propanoic acid	$C_6HF_{11}O_3$	330.05	13C3 HFPO-DA	
N-EtFOSAA	N-ethylperfluoro-1-octanesulfonamidoacetic acid	$C_{12}H_8F_{17}NO_4S$	585.23	d5 N-EtFOSAA	✓
N-MeFOSAA	N-methylperfluoro-1-octanesulfonamidoacetic acid	$C_{11}H_6F_{17}NO_4S$	571.21	d3 N-MeFOSAA	✓
PFBA	Perfluoro-n-butanoic acid	$C_4HF_7O_2$	213.98	13C4 PFBA	✓
PFBS	Potassium perfluoro-1-butanesulfonate	$C_4F_9O_3S.K$	338.19	13C3 PFBS	✓
PFDA	Perfluoro-n-decanoic acid	$C_{10}HF_{19}O_2$	514.08	13C6 PFDA	✓
PFDoA	Perfluoro-n-dodecanoic acid	$C_{12}HF_{23}O_2$	614.10	13C2 PFDoA	✓
PFDoS	Sodium perfluoro-1-dodecanesulfonate	$C_{12}F_{25}O_3S.Na$	722.14	13C8 PFOS	
PFHpA	Perfluoro-n-heptanoic acid	$C_7HF_{13}O_2$	364.06	13C4 PFHpA	✓
PFHpS	Sodium perfluoro-1-heptanesulfonate	$C_7F_{15}O_3S.Na$	472.10	13C3 PFHxS	✓
PFHxA	Perfluoro-n-hexanoic acid	$C_6HF_{11}O_2$	314.05	13C5 PFHxA	✓
PFHxS	Potassium perfluorohexanesulfonate	$C_6F_{13}O_3S.K$	438.20	13C3 PFHxS	✓
PFNA	Perfluoro-n-nonanoic acid	$C_9HF_{17}O_2$	464.08	13C9 PFNA	✓

Abbreviation	Compound Name	Molecular Formula	Molecular Weight	I.S.	Secondary Standard
PFNS	Sodium perfluoro-1-nonanesulfonate	$C_9F_{19}O_3S.Na$	572.12	13C8 PFOS	✓
PFOA	Perfluoro-n-octanoic acid	$C_8HF_{15}O_2$	414.07	13C8 PFOA	✓
PFOS	Potassium perfluorooctanesulfonate	$C_8F_{17}O_3S.K$	538.22	13C8 PFOS	✓
PFDS	Sodium Perfluoro-1-decanesulfonate	$C_{10}F_{21}SO_3.Na$	622.13	13C7 PFUdA	
PFPeA	Perfluoro-n-pentanoic acid	$C_5HF_9O_2$	264.05	13C5 PFPeA	✓
PFPeS	Sodium perfluoro-1-pentanesulfonate	$C_5F_{11}O_3S.Na$	372.08	13C3 PFHxS	✓
PFTeDA	Perfluoro-n-tetradecanoic acid	$C_{14}HF_{27}O_2$	714.11	13C2 PFTeDA	✓
PFTrDA	Perfluoro-n-tridecanoic acid	$C_{13}HF_{25}O_2$	664.11	13C7 PFUdA	✓
PFUdA	Perfluoro-n-undecanoic acid	$C_{11}HF_{21}O_2$	564.09	13C7 PFUdA	✓
PF5OHxA (or PFMBA)	Perfluoro-5-oxahexanoic acid	$C_5F_9O_3H$	279.98	13C5 PFHxA	
FPrPA (or 3:3 FTCA)	3-Perfluoropropyl propanoic acid	$C_6H_4F_7O_2H$	242.02	13C5 PFPeA	
PF4OPeA (or PFMPA)	Perfluoro-4-oxapentanoic acid	$C_4F_7O_3H$	229.98	13C5 PFPeA	
PFEESA	Perfluoro(2-ethoxyethane)sulfonate	$C_4F_9SO_4.K$	354.19	13C3 HFPO-DA	
3,6-OPFHpA (or NFDHA)	Perfluoro-3,6-dioxaheptanoic acid	$C_5HF_9O_4$	296.04	13C4 PFHpA	
FPePA (or 5:3 FTCA)	3-Perfluoropentyl propanoic acid	$C_8H_4F_{11}O_2H$	342.01	13C2-6-2FTS	
9Cl-PF3ONS	Potassium 9-chlorohexadecafluoro-3-oxanonane-1-sulfonate	$C_8F_{16}ClSO_4.K$	570.67	13C2-8-2FTS	
11Cl-PF3OUdS	Potassium 11-chloroeicosafluoro-3-oxaundecane-1-sulfonate	$C_{10}F_{20}ClSO_4.K$	670.69	d5-N-EtFOSAA	

Abbreviation	Compound Name	Molecular Formula	Molecular Weight	I.S.	Secondary Standard
N-MeFOSE	2-(N-Methylperfluoro-1-octanesulfonamido)ethanol	$C_{11}H_8F_{17}NO_3S$	616	d_7-N-MeFOSE-surrogate	
N-EtFOSE	2-(N-Ethylperfluoro-1-octanesulfonamido)ethanol	$C_{12}H_{10}F_{17}NO_3S$	630	d_9N-EtFOSE-surrogate	
N-MeFOSA	N-methylperfluoro-1-octanesulfonamide	$C_9H_4F_{17}NO_2S$	512.96	d N-MeFOSA-Surrogate	
N-EtFOSA	N-ethylperfluoro-1-octanesulfonamide	$C_{10}H_6F_{17}NO_2S$	526.98	d N-EtFOSA-Surrogate	
FHpPA (or 7:3 FTCA)	3-Perfluoroheptyl propanoic acid	$C_{10}H_4F_{15}O_2H$	442.01	M2-8-2FTS	
13C4-PFBA-Surrogate	Perfluoro-n-($^{13}C_4$)butanoic acid	$^{13}C_3F_7{}^{13}COOH$	217.99	13C3 PFBA	
13C5 PFPeA-Surrogate	Perfluoro-n($^{13}C_5$)-pentanoic acid	$^{13}C_4F_9{}^{13}COOH$	269.00	13C3 PFBA	
13C3 PFBS-Surrogate	Perfluoro-1-(2,3,4-$^{13}C_3$)butane sulfonic acid	$^{13}C_3{}^{12}CF_9SO_3H$	302.96	18O2 PFHxS	
13C2-4FTS-Surrogate	Sodium 1H, 1H, 2H, 2H-(1,2-$^{13}C_2$) 4:2 Fluorotelomer sulfonic acid	$^{13}C_2{}^{12}C_4F_9H_4SO_3.Na$	352.13	13C2 PFHxA	
13C5 PFHxA-Surrogate	Perfluoro-n-(1,2,3,4,6-$^{13}C_5$)hexanoic acid	$^{13}C_5{}^{12}CF_{11}O_2H$	318.99	13C2 PFHxA	
13C3 HFPO-DA-Surrogate	2,3,3,3-Tetrafluoro-2-(1,1,2,2,3,3,3-heptafluoropropoxy)- ($^{13}C_3$)propanoic acid	$^{13}C_3{}^{12}C_3HF_{11}O_3$	333.05	13C2 PFHxA	
13C4 PFHpA-Surrogate	Perfluoro-n-(1,2,3,4-$^{13}C_4$)heptanoic acid	$^{13}C_3{}^{12}C_3F_{13}{}^{13}COOH$	367.06	18O2 PFHxS	

Abbreviation	Compound Name	Molecular Formula	Molecular Weight	I.S.	Secondary Standard
13C3 PFHxS-Surrogate	Sodium perfluoro-1-(1,2,3-$^{13}C_3$)hexanesulfonate	$^{13}C_3{}^{12}C_3F_{13}O_3S.Na$	425.08	18O2 PFHxS	
13C2-6-2FTS-Surrogate	Sodium 1H,1H,2H,2H-perfluoro(1,2-$^{13}C_2$)octanesulfonate	$^{13}C_2{}^{12}C_6H_4F_{13}O_3S.Na$	452.13	13C4 PFOA	
13C8 PFOA-Surrogate	Perfluoro-n-($^{13}C_8$)octanoic acid	$^{13}C_8HF_{15}O_2$	422.00	13C4 PFOA	
13C8 PFOS-Surrogate	Potassium perfluoro-1-($^{13}C_8$)octanesulfonate	$^{13}C_8HF_{17}O_3S$	546.16	13C4 PFOS	
13C9 PFNA-Surrogate	Perfluoro-n-($^{13}C_9$)nonanoic acid	$^{13}C_9HF_{17}O_2$	473.08	13C5 PFNA	
13C2-8-2 FTS-Surrogate	Sodium 1H,1H,2H,2H-perfluoro(1,2-$^{13}C_2$)decanesulfonate	$^{13}C_2{}^{12}C_8H_5F_{17}O_3S$	529.98	13C2 PFDA	
13C6 PFDA-Surrogate	Perfluoro-n-(1,2,3,4,5,6-$^{13}C_6$)decanoic acid	$^{13}C_6{}^{12}C_4HF_{19}O_2$	519.99	13C2 PFDA	
13C7 PFUdA-Surrogate	Perfluoro-n-(1,2,3,4,5,6,7-$^{13}C_7$)undecanoic acid	$^{13}C_6{}^{12}C_4F_{21}{}^{13}COOH$	564.09	13C2 PFDA	
d3-N-MeFOSAA-Surrogate	N-methyl-d_3-perfluoro-1-octanesulfonamidoacetic acid	$C_{11}H_3D_3F_{17}NO_4S$	574.2	13C4 PFOA	
d5 N-EtFOSAA-Surrogate	N-ethyl-d_5-perfluoro-1-octanesulfonamidoacetic acid	$C_{12}H_3D_5F_{17}NO_4S$	590.2	13C4 PFOA	
13C2 PFDoA-Surrogate	Perfluoro-n-(1,2-$^{13}C_2$)dodecanoic acid	$^{13}C_2{}^{12}C_{10}HF_{23}O_2$	615.97	13C2 PFDA	
13C2 PFTeDA-Surrogate	Perfluoro-n-(1,2-$^{13}C_2$)tetradecanoic acid	$^{13}C_2{}^{12}C_{12}HF_{27}O_2$	715.96	13C2 PFDA	

Abbreviation	Compound Name	Molecular Formula	Molecular Weight	I.S.	Secondary Standard
13C8 FOSA-Surrogate	Perfluoro-1-($^{13}C_8$)octanesulfonamide	$^{13}C_8H_2F_{17}NO_2S$	499.14	13C2 PFDA	
d3-N-MeFOSA-Surrogate	N-methyl-d_3-perfluoro-1-octanesulfonamide	$C_9H_1D_3F_{17}NO_2S$	515.98	13C4 PFOS	
d_5-N-EtFOSA-Surrogate	N-ethyl-d_5-perfluoro-1-octanesulfonamide	$C_{10}H_1D_5F_{17}NO_2S$	532.01	13C4 PFOS	
d_7-N-MeFOSE-Surrogate	2-(N-Methyl-d_3-perfluoro-1-octanesulfonamido)ethan-d_4-ol	$C_{11}HD_7F_{17}NO_3S$	557.22	13C4 PFOS	
d_9-N-EtFOSE-Surrogate	2-(N-Ethyl-d_5-perfluoro-1-octanesulfonamido)ethan-d_4-ol	$C_{12}HD_9F_{17}NO_3S$	643.21	13C4 PFOS	
13C3 PFBA	Perfluoro-n-(2,3,4-$^{13}C_3$)butanoic acid	$^{13}C_3F_7COOH$	216.99		
13C2 PFHxA	Perfluoro-n-(1,2-$^{13}C_2$)hexanoic acid	$^{13}C_2{}^{12}C_4F_{11}O_2H$	315.99		
13C4 PFOA	Perfluoro-n-(1,2,3,4-$^{13}C_4$)octanoic acid	$^{13}C_4{}^{12}C_4HF_{15}O_2$	417.98		
13C5 PFNA	Perfluoro-n-(1,2,3,4,5-$^{13}C_5$)nonanoic acid	$^{13}C_5{}^{12}C_4HF_{17}O_2$	468.99		
13C2 PFDA	Perfluoro-n-(1,2-$^{13}C_2$)decanoic acid	$^{13}C_2{}^{12}C_8HF_{19}O_2$	515.97		
18O2 PFHxS	Sodium perfluoro-1-hexane($^{18}O_2$)sulfonate	$^{12}C_6F_{13}{}^{18}O_2OS.Na$	426.10		
13C4 PFOS	Potassium perfluoro-1-(1,2,3,4-$^{13}C_4$)octanesulfonate	$^{13}C_4{}^{12}C_4F_{17}O_3S.K$	538.22		

Table S3. Liquid chromatograph (LC) gradient conditions, using (A) 2mM ammonium acetate, and (B) methanol as the mobile phases.

Time (min)	A [%]	B [%]	Flow (mL/min)
0.00	90	10	0.4
10.00	5	95	0.4
13.00	5	95	0.4
15.00	90	10	0.4

Table S4. Mass spectrometer (MS) parameters for liquid chromatography (LC)-MS/MS analysis.

Parameter	Setting
MS Acquisition	Dynamic MRM
Cycle Time	500 ms
Ion Source	ESI negative
Drying Gas Temperature & Flow	230 °C & 6 L/min
Nebulizer	20 psi
Sheath Gas Temperature & Flow	355 °C & 10 L/min
Capillary	2500 V
Nozzle Voltage	0 V

Table S5. Summary of the dynamic Multiple Reaction Monitoring (dMRM) conditions for MS/MS analyses

Compound Group	Compound Name	Precursor Ion	Product Ion	RT (min)	Fragmentor (V)	Collision Energy(eV)	Cell Accelerator Voltage(V)
Acid	PFBA	213.0	168.9	3.5	60	8	2
Acid	PFPeA	263.0	219.0	4.5	72	4	2
Acid	PFHxA	313.0	268.9	5.2	70	8	2
Acid	PFHxA	313.0	119.0	5.2	70	18	2
Acid	PFHpA	362.9	319.0	5.7	72	4	2
Acid	PFHpA	362.9	169.0	5.7	72	14	2
Acid	PFOA	413.0	369.0	6.2	69	4	2
Acid	PFOA	413.0	169.0	6.2	69	12	2
Acid	PFNA	463.0	419.0	6.6	66	4	2
Acid	PFNA	463.0	219.0	6.6	66	17	2
Acid	PFDA	513.0	469.0	7.0	72	12	2
Acid	PFDA	513.0	219.0	7.0	72	20	2
Acid	PFUnA	563.0	519.0	7.5	100	12	2
Acid	PFUnA	563.0	269.0	7.5	100	20	2
Acid	PFDoA	613.0	569.0	7.9	100	8	2
Acid	PFDoA	613.0	319.0	7.9	100	20	2
Acid	PFTrDA	663.0	619.0	8.2	100	12	2
Acid	PFTrDA	663.0	169.0	8.2	100	32	2

Compound Group	Compound Name	Precursor Ion	Product Ion	RT (min)	Fragmentor (V)	Collision Energy(eV)	Cell Accelerator Voltage(V)
Acid	PFTeDA	712.9	669.0	8.6	100	12	2
Acid	PFTeDA	712.9	169.0	8.6	100	32	2
Ether sulfonic acids	PFEESA	314.9	134.9	5.6	110	24	2
Ether sulfonic acids	PFEESA	314.9	83.0	5.6	124	20	5
Ether sulfonic acids	PFEESA	314.9	69.0	5.6	110	60	2
Ether sulfonic acids	9Cl-PF3ONS	530.9	350.9	7.6	145	28	2
Ether sulfonic acids	9Cl-PF3ONS	530.9	83.0	7.6	145	32	2
Ether sulfonic acids	11Cl-PF3OUdS	630.9	83.0	8.4	160	32	2
Ether sulfonic acids	11Cl-PF3OUdS	630.9	450.9	8.4	165	32	2
Flurotelomer carboxylic acid	3-3 FTCA (FPrPA)	241.0	177.0	4.2	74	4	3
Flurotelomer carboxylic acid	3-3 FTCA (FPrPA)	241.0	117.0	4.2	74	44	3
Flurotelomer carboxylic acid	5-3 FTCA (FPePA)	341.0	237.0	5.6	84	12	3
Flurotelomer carboxylic acid	5-3 FTCA (FPePA)	341.0	217.0	5.6	84	24	3
Flurotelomer carboxylic acid	7-3 FTCA (FHpPA)	441.0	337.0	6.6	76	12	3
Flurotelomer carboxylic acid	7-3 FTCA (FHpPA)	441.0	317.0	6.6	76	24	3
FTS	4:2FTS	327.0	306.9	5.0	125	20	2
FTS	4:2FTS	327	80.9	5.0	125	36	2
FTS	6:2FTS	427.0	406.8	6.0	125	24	2
FTS	6:2FTS	427.0	80.9	6.0	125	40	2
FTS	8:2FTS	527.0	507.0	6.8	200	30	4
FTS	8:2FTS	527.0	80.9	6.8	170	40	2
ISTD	13C3-PFBA	216.0	171.9	3.5	65	8	2
ISTD	13C2-PFHxA	315.0	270.0	5.2	70	8	2
ISTD	13C4-PFOA	417.0	172.0	6.2	69	12	2
ISTD	18O2-PFHxS	403.0	83.9	6.4	100	49	2
ISTD	13C5-PFNA	468.0	423.0	6.6	66	4	2
ISTD	13C2-PFDA	515.0	470.0	7.0	81	4	2
ISTD	13C4-PFOS	503.0	80.0	7.3	148	54	2
ISTD	13C4-PFOS	502.9	98.9	7.3	180	48	2
Perfluorooctane sulfonamide ethanols	NMeFOSE	616.0	59.0	9.8	82	15	4
Perfluorooctane sulfonamide ethanols	NEtFOSE	630.0	59.0	10.1	124	45	4
Perfluorooctane sulfonamides	FOSA	497.9	478.0	8.6	150	36	3
Perfluorooctane sulfonamides	FOSA	497.9	78.0	8.6	150	36	3

Compound Group	Compound Name	Precursor Ion	Product Ion	RT (min)	Fragmentor (V)	Collision Energy(eV)	Cell Accelerator Voltage(V)
Perfluorooctane sulfonamides	FOSA	497.9	48.0	8.6	150	110	3
Perfluorooctane sulfonamides	NMeFOSA	512.0	219.0	9.9	156	28	5
Perfluorooctane sulfonamides	NMeFOSA	512.0	169.0	9.9	156	32	5
Perfluorooctane sulfonamides	NEtFOSA	526.0	269.0	10.3	160	28	5
Perfluorooctane sulfonamides	NEtFOSA	526.0	219.0	10.3	160	28	5
Perfluorooctane sulfonamides	NEtFOSA	526.0	169.0	10.3	160	28	5
Perfluorooctane sulfonamidoacetic acids	NMeFOSAA	570.0	482.9	7.1	150	16	2
Perfluorooctane sulfonamidoacetic acids	NMeFOSAA	570.0	419.0	7.1	150	20	2
Perfluorooctane sulfonamidoacetic acids	NEtFOSAA	584.0	526.0	7.3	100	20	2
Perfluorooctane sulfonamidoacetic acids	NEtFOSAA	584.0	419.0	7.3	100	20	2
Polyfluoroether carboxylic acids	PFMPA (PF4OPeA)	229.0	84.9	4.0	60	12	2
Polyfluoroether carboxylic acids	PFMBA (PF5OHxA)	279.0	235.0	4.8	80	1	5
Polyfluoroether carboxylic acids	PFMBA (PF5OHxA)	279.0	84.9	4.8	70	12	2
Polyfluoroether carboxylic acids	NFDHA (3,6 OPFHpA)	295.0	201.0	5.1	75	5	2
Polyfluoroether carboxylic acids	NFDHA (3,6 OPFHpA)	295.0	85.0	5.1	120	32	5
Polyfluoroether carboxylic acids	NFDHA (3,6 OPFHpA)	201.0	85.0	5.1	70	15	5
Polyfluoroether carboxylic acids	HFPO-DA	285.0	185.0	5.4	50	20	5
Polyfluoroether carboxylic acids	HFPO-DA	285.0	169.0	5.4	50	4	5
Polyfluoroether carboxylic acids	ADONA	377.0	250.9	5.9	80	12	2
Polyfluoroether carboxylic acids	ADONA	377.0	85.0	5.9	80	36	2
Sulfonate	PFBS	298.9	98.9	5.3	100	29	2
Sulfonate	PFBS	298.9	80.0	5.3	100	45	2
Sulfonate	PFPeS	348.9	98.9	5.9	135	40	2
Sulfonate	PFPeS	348.9	79.9	5.9	135	40	2
Sulfonate	PFHxS	398.9	99.0	6.4	100	45	2

Compound Group	Compound Name	Precursor Ion	Product Ion	RT (min)	Fragmentor (V)	Collision Energy(eV)	Cell Accelerator Voltage(V)
Sulfonate	PFHxS	398.9	80.0	6.4	100	49	2
Sulfonate	PFHpS	448.9	98.7	6.9	100	44	2
Sulfonate	PFHpS	448.9	80.0	6.9	148	50	2
Sulfonate	PFOS	498.9	99.0	7.3	100	50	2
Sulfonate	PFOS	498.9	80.0	7.3	100	50	2
Sulfonate	PFNS	548.9	99.0	7.7	148	52	2
Sulfonate	PFNS	548.9	80.0	7.7	148	56	2
Sulfonate	PFDS	598.9	99.0	8.1	148	56	2
Sulfonate	PFDS	598.9	80.0	8.1	148	60	2
Sulfonate	PFDoS	698.9	99.0	8.8	156	62	2
Sulfonate	PFDoS	698.9	80.0	8.8	156	67	2
Surrogate	13C4-PFBA	217.0	172.0	3.5	60	8	2
Surrogate	13C5-PFPeA	268.0	223.0	4.5	60	8	2
Surrogate	13C2-4:2FTS	329.0	309.0	5.0	125	20	2
Surrogate	13C2-4:2FTS	329.0	81.0	5.0	150	32	2
Surrogate	13C5-PFHxA	318.0	273.0	5.2	70	8	2
Surrogate	13C5-PFHxA	318.0	120.0	5.2	72	24	2
Surrogate	13C3-PFBS	302.0	99.0	5.3	130	32	2
Surrogate	13C3-PFBS	302.0	80.0	5.3	130	44	2
Surrogate	13C3-HFPO-DA	287.0	185.0	5.4	64	20	5
Surrogate	13C3-HFPO-DA	287.0	169.0	5.4	64	4	5
Surrogate	13C4-PFHpA	367.0	322.0	5.7	72	4	2
Surrogate	13C4-PFHpA	367.0	169.0	5.7	72	16	2
Surrogate	13C2-6:2FTS	429.0	409.0	6.0	125	24	2
Surrogate	13C2-6:2FTS	429.0	81.0	6.0	150	40	2
Surrogate	13C8-PFOA	421.0	376.0	6.2	69	4	2
Surrogate	13C8-PFOA	421.0	172.0	6.2	72	20	2
Surrogate	13C3-PFHxS	402.0	99.0	6.4	156	44	2
Surrogate	13C3-PFHxS	402.0	80.0	6.4	100	45	2
Surrogate	13C9-PFNA	472.0	427.0	6.6	66	4	2
Surrogate	13C9-PFNA	472.0	223.0	6.6	72	16	2
Surrogate	13C2-8:2FTS	529.0	509.0	6.8	170	28	2
Surrogate	13C2-8:2FTS	529.0	81.0	6.8	200	52	4
Surrogate	13C6-PFDA	519.0	474.0	7.0	72	8	2
Surrogate	D3-NMeFOSAA	573.2	419.0	7.1	150	20	2
Surrogate	D5-NEtFOSAA	589.2	419.0	7.2	100	20	2
Surrogate	13C8-PFOS	507.0	99.0	7.3	148	52	2
Surrogate	13C8-PFOS	507.0	80.0	7.3	100	50	2
Surrogate	13C7-PFUnA	570.0	525.0	7.5	100	8	2

Compound Group	Compound Name	Precursor Ion	Product Ion	RT (min)	Fragmentor (V)	Collision Energy(eV)	Cell Accelerator Voltage(V)
Surrogate	13C2-PFDoA	615.0	570.0	7.9	90	12	2
Surrogate	13C2-PFTeDA	715.0	670.0	8.6	90	12	2
Surrogate	13C8-PFOSA	506.0	78.0	8.6	150	36	3
Surrogate	D7-NMeFOSE	623.2	59.0	9.8	82	15	4
Surrogate	D3-NMeFOSA	515.0	219.0	9.9	156	28	5
Surrogate	D9-NEtFOSE	639.2	59.0	10.1	124	45	4
Surrogate	D5-NEtFOSA	531.0	219.0	10.2	160	28	5

Note: *In accordance with US EPA Method 1633, two MRM transitions were monitored for each PFAS when feasible. Some short-chain PFAS (e.g., PFBA, PFPeA) produce only one reliable product ion due to limited fragmentation. In these cases, compound identity was confirmed using retention time matching, isotope-dilution internal standards, and required quality control criteria, consistent with Method 1633.*

Table S6. Method performance for detected PFAS in this study.

Compounds	IDL (ng/L)	MDL (ng/L)	Recoveries (%) ± SD (n=4)	Surrogate Recoveries (%) (n=12)
PFBA	8	0.09	99.60 ± 10.80	48.80 -111.00
PF4OPeA	4	0.04	55.10 ± 19.40	N/A
PFPeA	4	0.04	95.90 ± 2.85	92.00-164.00
PFBS	2	0.02	108.00 ± 3.88	41.90-131.00
PFHxA	2	0.02	106.00 ± 4.07	60.50-102.00
GenX	4	0.04	113.00 ± 3.28	56.50-141.00
PFPeS	5	0.06	83.90 ± 20.80	N/A
PFHxS	2	0.02	116.00 ± 7.76	50.90-102.00
PFHpA	2	0.02	93.90 ± 13.30	45.30-107.00
6:2 FTS	40	0.44	119.00 ± 10.20	52.10-195.00
PFHpS	5	0.06	85.50 ± 10.20	N/A
PFOA	2	0.02	117.00 ± 3.89	53.90-105.00
PFOS	5	0.06	90.80 ± 50.00	45.70-71.90
PFNA	5	0.06	96.50 ± 6.49	54.10- 98.30
8:2 FTS	20	0.22	77.60 ± 5.44	41.40- 156.00
PFDA	10	0.11	111.00 ± 31.90	37.00-87.80
PFDS	10	0.11	29.20 ± 0.88	N/A
N-MeFOSAA	5	0.06	129.20 ± 4.42	37.10-205.00
N-EtFOSAA	10	0.11	79.80 ± 13.60	66.00-158.00
PFDoA	20	0.22	117.00 ± 3.81	36.20- 160.00
FOSA	5	0.06	116.00 ± 13.40	25.20-142.00

IDL: instrument detection limit; MDL: method detection limit; SD: standard deviation; N/A: not available.
A concentration of 500 ng/L was used to estimate the recovery

Table S7. Gradient Conditions for the Orbitrap

**Time (min)**	**[A]%**	**[B]%**	**Flow rate (mL min⁻¹)**
**0.00**	100	0	0.40
**1.00**	70	30	0.40
**6.00**	55	45	0.40
**13.00**	20	80	0.40
**14.00**	5	95	0.40
**17.50**	5	95	0.40
**18.00**	100	0	0.40
**20.00**	100	0	0.40

A: 2 mM ammonium acetate in H_2O:MeOH (95:5, v/v) **B:** MeOH

Column oven: 50 °C **Total run time:** 20.0 min

Table S8. MS parameters for Full MS and data-dependent MS (ddMS2)

**General**	
Runtimes	0-20 min
Polarity	Negative
**Full MS**	
Resolution	120,000
Scan range	100 to 800 *m/z*
AGC target	3e6
Maximum IT	50ms
**dd-MS2/dd**	
Resolution	> 80,000
Scan Range (*m/z*)	50–800
AGC target	Standard
Maximum IT	50 ms
Loop counts	5
Isolation window	1.3 *m/z*
HCD Collision Energies (%)	30
Detector Type	Astral

Table S9. $ddMS^2$ Trigger and Exclusion Parameters

**Parameter**	
Intensity filter type	IntensityThreshold
Minimum intensity for MS/MS	250,000
Mass list type	*m/z*
Include intensity threshold	False
Time mode	Unscheduled
Mass tolerance type	ppm
Mass tolerance (high)	±10 ppm
Mass tolerance (low)	±10 ppm
Ignore charge state requirement	True
Set activation per compound	False
Perform dependent scan on most intense ion if no targets found	False

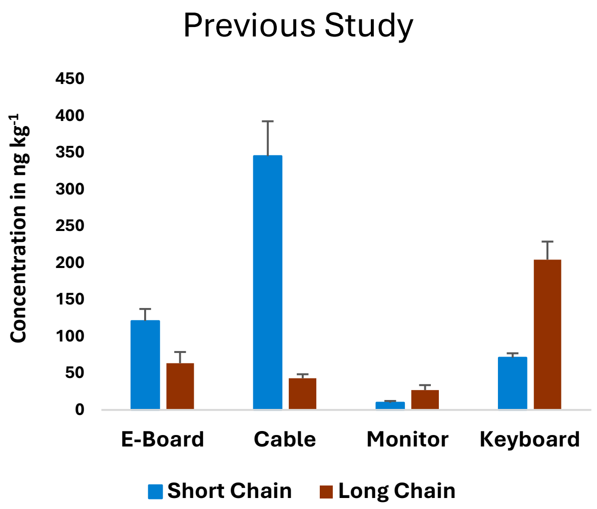

Figure S1. Distribution of short- and long-chain PFAS across e-waste components *(number of samples n = 2 independent composite replicates, each comprising ≥4 items)*.

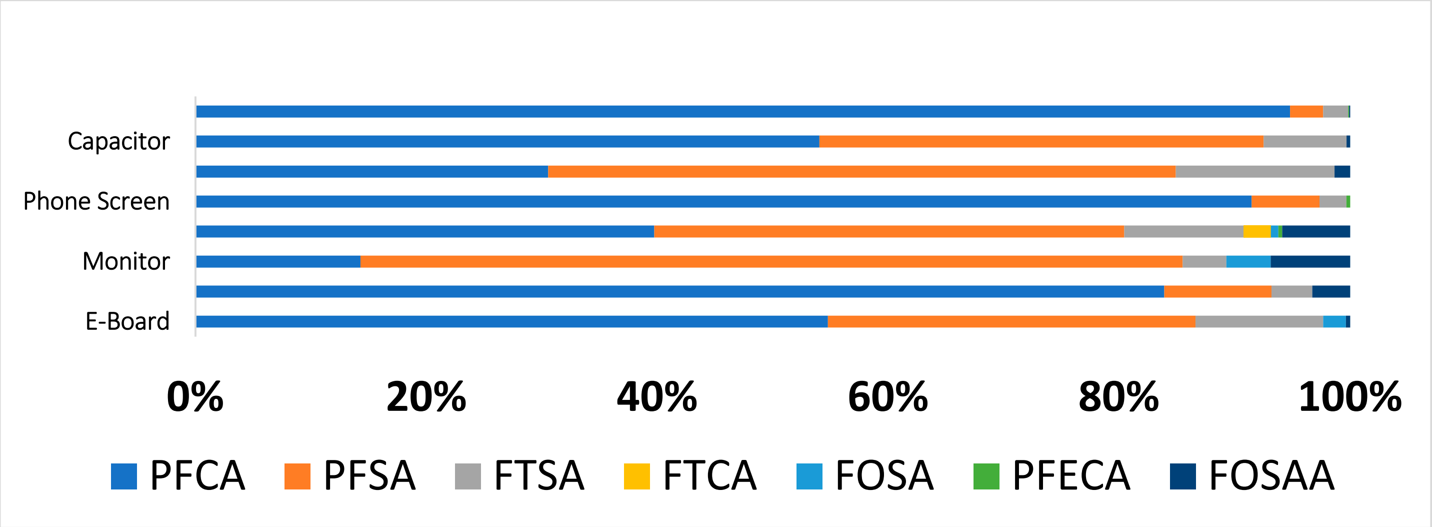

Figure S2. Proportion of PFAS classes in the e-waste leachate. *(number of samples n = 2 independent composite replicates, each comprising ≥4 items)*

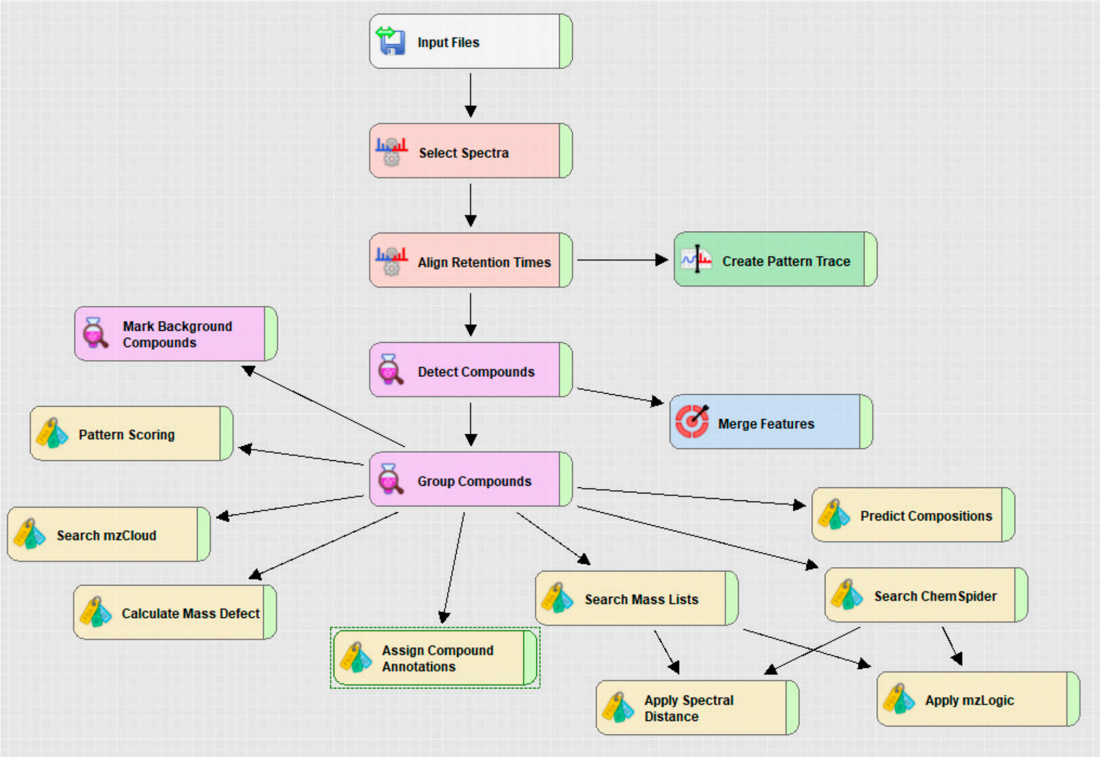

Figure S3. Compound Discovered workflow for the screening of PFAS.
